# Urocortin suppresses endometrial cancer cell migration via CRFR2 and its system components are differentially modulated by estrogen

**DOI:** 10.1002/cam4.967

**Published:** 2017-01-21

**Authors:** Gemma L. Owens, Kevin M. Lawrence, Tom R. Jackson, Emma J. Crosbie, Berna S. Sayan, Henry C. Kitchener, Paul A. Townsend

**Affiliations:** ^1^ Division of Molecular & Clinical Cancer SciencesSchool of Medical SciencesFaculty of BiologyMedicine & HealthManchester Cancer Research CentreUniversity of ManchesterWilmslow RoadManchesterM20 4QLUnited Kingdom; ^2^ Division of Molecular & Clinical Cancer SciencesSchool of Medical SciencesFaculty of BiologyMedicine & HealthSt Mary's HospitalUniversity of ManchesterOxford RoadManchesterM13 9WLUnited Kingdom

**Keywords:** CRF receptors, endometrial cancer, estrogen receptor, urocortin

## Abstract

Urocortin (UCN1) peptide shares structural and functional homology with corticotropin‐releasing factor (CRF). UCN1 is significantly reduced in endometrial adenocarcinoma compared to healthy controls. However, there are no data which evaluate the effects of UCN1 in the endometrium, or how it is modulated. We used proliferation and transwell assays to determine the effect of UCN1 on the proliferation and migration of Ishikawa and HEC1A cells. We also determined the expression levels of UCN1 and its receptors produced by estrogen receptor agonists, and the effect of UCN1 on estrogen receptor expression, using quantitative polymerase chain reaction. UCN1 suppressed migration of endometrial cancer cells in vitro. This effect appears to be specific to CRF receptor 2 (CRFR2), as selective antagonism of CRFR2 but not CRFR1 completely eliminated suppression of migration. Activation of ERA reduced UCN1 expression, but only had a small effect on the expression of CRFR1. However, expression of CRFR2 was more notably reduced at both the mRNA and protein levels by activation of ERB. UCN1 in turn reduced both ERA and ERB expression, as assessed by real‐time quantitative PCR. We demonstrate that UCN1 significantly suppresses the migration of endometrial cancer cells but has no effect on their proliferation. Thus, loss of UCN1 in endometrial cancer may promote invasion and metastatic spread. There is a complex relationship between the UCN1 system and estrogen receptors, which may provide insights into endometrial carcinogenesis, a disease known to be driven by estrogen excess.

## Introduction

Endometrial cancer is the most common gynecological malignancy in developed countries, with 320,000 new cases diagnosed worldwide in 2012 1. The incidence of endometrial cancer has increased by 40% in the last two decades, and this has largely been attributed to the increased prevalence of obesity and the use of uterine‐sparing treatments in benign gynecology 2. The mainstay of treatment for endometrial cancer is surgery; however, this can be technically challenging in obese and elderly patients. Consequently, there has been a growing interest in scrutinizing the molecular pathways involved in endometrial carcinogenesis, to enable the development of novel medical therapies.

Urocortin 1 (UCN1) is a 40‐amino acid peptide, which shares structural and functional homology with corticotropin‐releasing factor (CRF) 3. It has previously been shown that CRF suppresses proliferation and counteracts estradiol‐induced proliferation in the Ishikawa endometrial adenocarcinoma cell line, via its receptor, CRFR1 4, 5. In view of the homology shared between UCN1 and CRF, it was hypothesized that UCN1 may also be implicated in endometrial carcinogenesis. Indeed, a study by Florio et al. 6. found that UCN1 mRNA and peptide expression was significantly decreased in endometrial adenocarcinoma compared to healthy postmenopausal controls. The authors subsequently postulated that reduced expression of UCN1 might promote carcinogenesis through a lack of UCN‐mediated inhibition of cell proliferation. However, to date, there is no published data that evaluated the local effects of UCN1 in endometrial cancer.

Similarly, little is known with regard to how UCN1 is modulated in the endometrium. In benign endometrium, there is little expression in the stroma and abrupt changes in glandular expression following the menopause, which coincide with profound changes in ovarian steroid hormone synthesis 7. This suggests that UCN1 expression may be influenced by hormonal status. Indeed, in the malignant state, estrogen has been shown to decrease the activity of the CRF promoter via a direct effect on the functional estrogen response elements of the promoter region of the CRF gene 8.

In this study, we investigated the effect of UCN1 on endometrial cancer cell proliferation and migration, and the impact of estrogen on its expression.

## Materials and Methods

### Cell culture

Ishikawa cells were maintained in Eagle Minimum Essential Medium with L‐glutamine (Gibco), supplemented with 5% fetal calf serum (FCS) and 1% penicillin/streptomycin (Gibco) in 5% CO_2_/95% humidity at 37°C. HEC1A cells were maintained in McCoy 5A with L‐glutamine (Gibco), supplemented with 10% FCS and 1% penicillin/streptomycin under the same conditions. Cells were passaged every 2–3 days.

### Proliferation assay

Ishikawa cells were seeded in 24‐well culture plates at a density of 5000 cells per well. After incubation at 37°C for 24 h, culture medium was replaced by medium containing 100 nmol/L of UCN1 in triplicate wells and fresh culture medium in triplicate wells. 24, 48, 72, and 96 h after treatment, cell cultures were washed with PBS and fixed with 1 mL of ice‐cold fixative (50% methanol and 50% acetone) and stored in PBS until stained. Cells were stained with DAPI, and the center field of each well was photographed using a fluorescent microscope. The average cell number was counted using a cell counting macro on ImageJ software. The experiment was repeated three times.

### Transwell migration assays

Endometrial cancer cell migration was determined using Transwell chambers with 8‐*μ*m sized pores (BD Biosciences). Ishikawa and HEC1A cells were cultured in medium containing FCS for 24 h. After 24‐hour incubation, cells were re‐suspended. A quantity of 850 *μ*L of normal medium was placed into each well of a 24‐well plate and 350 *μ*L of cells in normal medium (250,000 cells/350 *μ*L) were loaded into the transwell inserts and placed at 37°C for 2 h to allow the cells to adhere to the inserts. The medium was then removed from the inserts and replaced with 350 *μ*L of serum‐free medium ±100 nmol/L of human UCN1. Cells were returned to the incubator. Cells were fixed at 24, 48, and 72 h using an ice‐cold fixative made with 50% methanol and 50% acetone, and stored in PBS until stained. Cells that had not penetrated through the filter were stained with eosin and wiped out with a cotton tip, and cells that had migrated to the bottom of the filter were stained with DAPI, and counted using a fluorescent microscope. The average cell number was counted using the center field of each sample. The experiment was repeated three times. The number of cells, which penetrated the Transwell chamber in each group, determined migration.

UCN1 binds with different affinities to two receptors, CRF receptor ‐1 (CRFR1) and ‐2 (CRFR2) 9, 10. To determine if the observed effect on cell migration was receptor specific, the above experiment was repeated with the use of selective antagonists to CRFR1 (CP154526) and CRFR2 (Astressin 2B), and using a non‐selective antagonist (alpha‐helical CRH) in addition to UCN1. Inhibitors were added 60 min prior to treatment with UCN1, at a concentration of 100 nmol/L each.

### Drug treatments for PCR and western blot experiments

Cell cultures were treated with 100 nmol/L UCN1, 40 nmol/L of PPT, a selective agonist for ER*A* or 50 nmol/L of ERB‐041, a selective agonist for ER*B* (all from Tocris Bioscience), and incubated for 24 h before harvesting.

### RNA isolation, semi‐quantitative and quantitative RT‐PCR

Total cellular RNA was isolated using the RNAeasy MiniKit (Qiagen) and treated with RNase‐free DNase (Qiagen), according to the manufacturer's instructions. RNA purity and concentration was determined using the NanoDrop 2000c Spectrophotometer (NanoDrop Technologies) at 260 and 280 nm. RNA integrity was confirmed by 1% agarose gel electrophoresis in 1× Tris‐Acetate‐EDTA buffer, with GelRed nucleic acid stain (Biotium) and visualization under UV transilluminator. cDNA was synthesized from 2 *μ*g of each RNA sample using the Omniscript reverse transcription kit (Qiagen), as per the manufacturer's instructions. Briefly, RNA was initially denatured with Oligo‐dT mix at 65°C for 10 min. Following the addition of the other components, reverse transcription was performed at 37°C for 60 min.

Semi‐quantitative RT‐PCR was performed to assess the effect of estrogen treatment on the expression of CRFR1 and CRFR2 transcripts in Ishikawa endometrial cancer cells using the GeneAmp PCR System 9700 (Applied Biosystems) and 2× MyTaq Red PCR Mastermix. The PCR primer sequences and conditions are listed in Table 1. PCR products were analyzed by agarose gel electrophoresis.

**Table 1:** Primer sequences and PCR protocol for semi‐quantitative PCR

Target Gene	Sense Primer Sequence (5′ → ‘3)	Anti‐Sense Primer Sequence (5′ → 3′)	Length of fragment (bp)	PCR protocol	Cycles
CRFR1	AGGGGCCTCTGGCTTCCC	CGTCGTGCGTACAGGGACGT	100	95°C/62°C/60°C	30
CRFR2	CAGACGGCCGCTGTGTGACC	ACACGACCCGTCCGAGAGCA	100	95°C/60°C/60°C	30
GAPDH	CCTGCTTCACCACCTTCTTG	CATCATCTCTGCCCCCTCTG	437	95°C/58°C/60°C	30

CRFR, corticotropin‐releasing factor receptor.

Quantitative RT‐PCR was performed to assess the expression of UCN1, ER*A*, and ER*B* transcripts in Ishikawa endometrial cancer cells using a Stratagene MX3000 and Precision 2× Real‐Time PCR Mastermix for Stratagene MX (PrimerDesign). The target sequences were amplified using customized primers for use with SYBR green (PrimerDesign). Internal reference genes were determined in advance using a geNorm kit (PrimerDesign) containing a panel of six candidate genes (GAPDH, B2M, UBC, TOP1, ACTB, and RPL13). Primer sequences are given in Table 2. Each reaction was performed in triplicate in 96‐well plates (PrimerDesign) under the following thermocycling conditions given in Table 2. No template controls were performed in triplicate for each target gene, and no amplification was produced. Results were analyzed using Biogazelle software.

**Table 2:** Primer sequences and PCR protocol for quantitative RT‐PCR

Target Gene	Forward Primer Sequence (5′ → ‘3)	Length of fragment (bp)	PCR protocol	Cycles
UCN	AGACCCTGTGTTCCCCAAG	79	95°C/95°C/60°C	40
ER*α*	AAGAAAGAACAACATCAGCAGTAAA	135	95°C/95°C/60°C	40
ER*β*	GCACGGCTCCATATACATACC	117	95°C/95°C/60°C	40

UCN, urocortin 1.

### Western blot analysis

Total cell lysates were collected after 24‐h incubation with the relevant drug treatments. 20 *μ*g of protein samples were separated by 10 or 12% SDS‐polyacrylamide gel electrophoresis run at 100V for 90 min and transferred onto polyvinylidene fluoride (PVDF) membranes. Membranes were blocked in 5% non‐fat milk solution in PBS for 1 h, to prevent nonspecific binding. Following this, membranes were incubated in primary antibodies overnight at 4°C.

Primary antibodies and the concentrations used were as follows: anti‐UCN1 mouse antibody (1:1000, R&D Systems), anti‐ER*A* and ER*B* rabbit antibody (1:1000,Sigma‐Aldrich), anti‐CRFR1 and anti‐CRFR2 rabbit antibodies (1:1000, Sigma‐Aldrich). Antibodies were diluted in 5% nonfat milk in PBS containing 0.1% Tween‐20. Following incubation with primary antibody, membranes were washed in PBS containing 0.1% Tween‐20 before the addition of a secondary goat anti‐rabbit or rabbit anti‐mouse HRP‐conjugated antibody (1:10,000). Specific antibody binding was detected by enhanced chemiluminescence (Pierce Biotechnology). Anti‐GAPDH mouse and anti‐*β*‐tubulin rabbit antibodies (1:1000, abcam) were used as loading controls. Densitometry analysis was performed using Image J software.

### Statistical analysis

Data presented in the figures are expressed as means ± SEM of three independent experiments. Differences between groups were evaluated using an independent unpaired t‐test. Statistical significance was assumed when *P* ≤ 0.05. Statistical tests were performed using Microsoft Excel or GraphPad Prism software, as applicable.

## Results

### UCN1 has no significant effect on endometrial cancer cell proliferation

**Figure 1:**
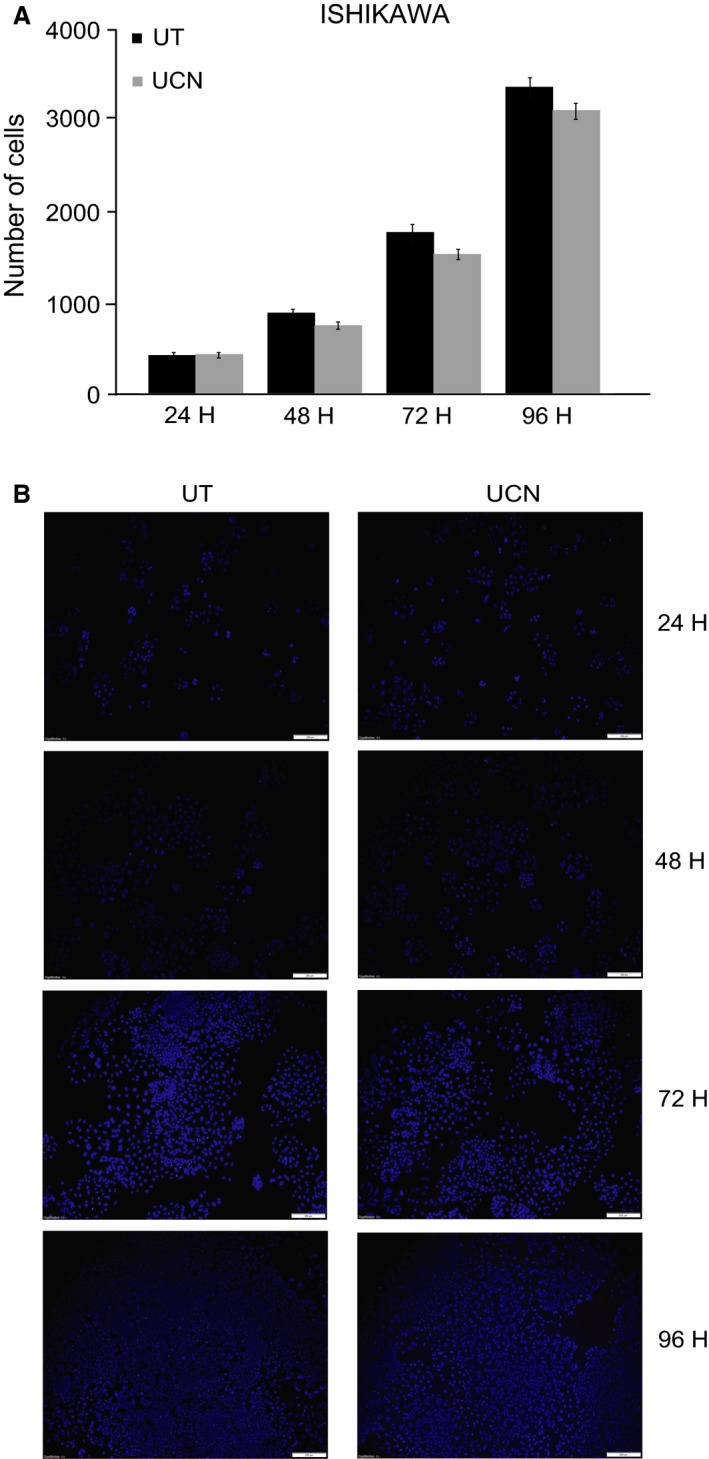
UCN1 treatment of Ishikawa cells has no significant effect on cell proliferation. Ishikawa cells were treated with 100 nmol/L UCN1 and cell proliferation was assessed at the indicated time periods. Cells were fixed and stained with DAPI and proliferation determined by enumeration of DAPI‐stained cell nuclei. (A) The average number of cells in untreated and UCN1‐treated wells. Each error bar represents the mean ± SEM of experiments performed in triplicate and repeated three times. (UT = untreated, UCN = urocortin1). (B) Representative images of DAPI‐stained cell cultures under the fluorescent microscope. Scale bar: 400 *μ*m.

To evaluate whether UCN1 has an effect on growth of endometrial cancer in vitro, Ishikawa cell cultures were treated with 100 nmol/L of UCN1, then fixed and stained with DAPI at 24, 48, 72, and 96 h. Proliferation was determined by enumeration of DAPI‐stained cell nuclei. As shown in Figure 1A and B, UCN1 treatment had no significant effect on cell proliferation at any of the given time points.

### UCN1 suppresses endometrial cancer migration via CRFR2

**Figure 2:**
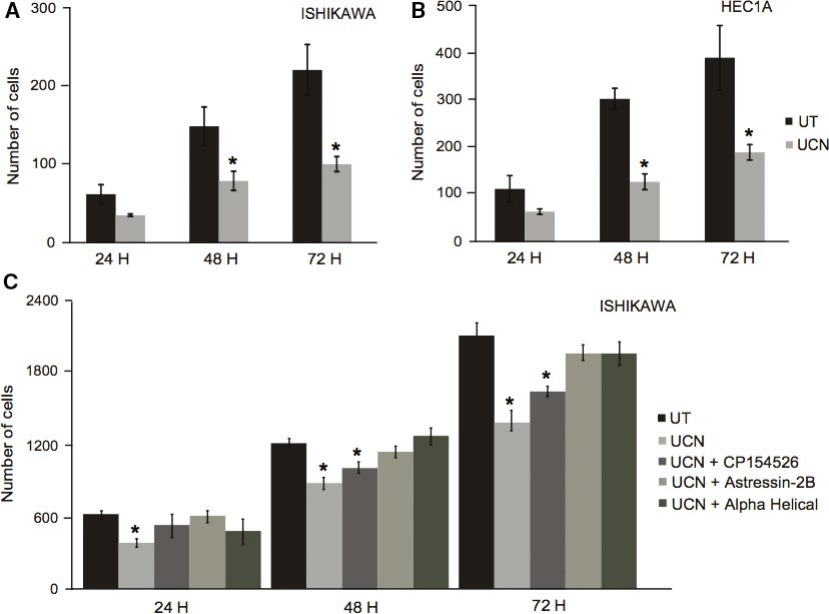
UCN1 treatment significantly suppresses migration of endometrial cancer cells via corticotropin‐releasing factor receptor 2 (CRFR 2). Ishikawa and HEC1A cells were seeded into transwell chambers at a known density, and treated with 100 nmol/L of UCN1 ±  CRF receptor antagonists for 24, 48, and 72 h. Transwell chambers were then fixed and stained. Migration was determined by counting the cells that had penetrated the transwell filter. (A) and (B) shows the average number of Ishikawa and HEC1A cells penetrating triplicate transwell chambers, respectively. Results indicate that UCN1 significantly suppressed Ishikawa cell migration at 48 and 72 h. (C) shows that UCN1 treatment significantly suppresses migration of Ishikawa cells via CRFR2. Ishikawa cells were treated with 100 nmol/L UCN1 ± 100 nmol/L of CP154526 (CRFR1 antagonist), Astressin‐2B (CRFR2 antagonist) or alpha‐helical (non‐selective CRFR antagonist) for 24, 48, and 72 h. Antagonism of CRFR1 led to significantly reduced migration at 48 and 72 h; however, antagonism of CRFR2 reversed this effect. Each bar represents the mean ± SEM of three independent experiments (*P < 0.05 calculated by Student's t‐test; UT = untreated, UCN = urocortin1).

Ishikawa and HEC1A cells were treated with 100 nmol/L of UCN1 and transwell migration assays were performed. The number of cells that penetrated the transwell membrane after 24, 48, and 72 h were counted to determine migration. Our results clearly indicate that UCN1 treatment significantly suppressed migration of both Ishikawa and HEC1A cells compared with negative controls, at 48 and 72 h (*P* < 0.05) (Fig. 2A and B).

To investigate the role of the CRF receptor subtypes in cell migration, transwell migration assays were repeated, with the addition of selective antagonists to CRFR1 (CP154526) and CRFR2 (Astressin 2B), and a non‐selective antagonist (alpha‐helical CRH). Cell cultures were treated with inhibitors for 60 min prior to the addition of UCN1. Our results indicate that UCN1 suppresses cell migration via CRFR2, as selective antagonism of CRFR1 significantly reduced migration at 48 and 72 h (*P* < 0.05) (Fig. 2C). Selective antagonism of CRFR2 reversed this effect. Antagonism of CRFR1 and CRFR2 simultaneously using alpha‐helical, appeared to have no significant effect on the rate of cell migration.

### Estrogen perturbs expression of UCN1 and the CRF receptors

**Figure 3:**
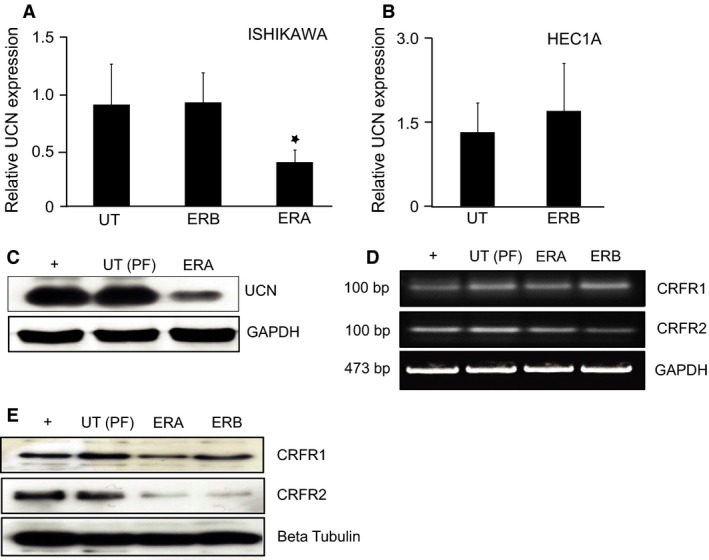
Activation of estrogen receptor alpha (ER*A*) down‐regulates UCN1 expression. (A and B) qPCR analysis on UCN1 expression in untreated cells and cells treated with selective agonists to ER*A* and ER*B*. HEC1A cells are ER*A* negative; therefore, the effect of ER*A* activation was not applicable. Each bar represents the mean ± SEM of triplicate samples, and each experiment was repeated three times (*P < 0.05, calculated by Student's t‐test, UT = untreated, ER*A* = ER*A* agonist, ER*B* = ER*B* agonist). (C) Western‐blot analysis of UCN1 expression in untreated and ERA‐treated Ishikawa cells. UCN1 protein expression was determined using mouse anti‐UCN1 monoclonal antibody, with a mouse anti‐GADPH monoclonal antibody used as a loading control. Bands with sizes corresponding to 5 kDa (UCN) and 37 kDa (GADPH) were detected. (D) Semi‐quantitative RT‐PCR of untreated, ER*A* *‐* and ER*B*‐treated Ishikawa cells. Predicted amplicon sizes are indicated. GAPDH was used as a loading control. (E) Western‐blot analysis of corticotropin‐releasing factor receptor 1 (CRFR1) and CRFR2 expression in untreated, ER*A* *‐* and ER*B*‐treated cells. CRFR1 and CRFR2 protein expression were determined using rabbit anti‐CRFR1 and rabbit anti‐CRFR2 monoclonal antibodies, with mouse anti‐*β*‐tubulin monoclonal antibody used as a loading control. Bands with sizes corresponding to 50 kDa (CRFR1), 47 kDa (CRFR2), and 50 kDa (*β*‐tubulin) were detected. Human chondrocytes were used as a positive control (+) for both (C), (D) and (E).

Ishikawa and HEC1A cells were cultured in estrogen‐free phenol red‐free medium containing charcoal stripped FCS and treated with selective estrogen receptor agonists to ER*A* (PPT) and ER*B* (ERB‐041). After 24 h incubation, cells were harvested and RNA and protein were isolated. Quantitative real‐time PCR was performed to compare the expression of UCN1 in response to estrogen receptor activation. Optimum annealing temperature and amplification cycle number were determined by PrimerDesign (Optimum conditions and primer sequences shown in Table 1). Verification of priming specificity was carried out by dissociation curve analysis. No template controls were performed alongside DNA samples to ensure that no PCR product contamination had occurred. UCN1 expression was normalized to internal reference genes selected in advance from a panel of six candidate genes, using GeNorm. Basal expression of UCN1 was determined in control (phenol red free/charcoal stripped FCS, untreated cells) and compared with cells treated with ER*A* and ER*B* agonists. Figure 3 clearly indicates that UCN1 mRNA expression is down regulated in response to activation of ER*A* (*P* < 0.05), whereas activation of ER*B* has no significant effect on UCN expression in either Ishikawa (Fig. 3A) or HEC1A cells (Fig. 3B). HEC1A cells do not express ER*A*; thus, the effects of an ER*A* agonist of UCN1 expression was not investigated in this cell line.

To determine whether ER*A* activation also down‐regulated UCN1 protein expression, western blot analysis with a primary antibody specific to UCN1 was performed. Figure 3C shows UCN1 protein expression is also significantly reduced in ER*A* agonism of Ishikawa cells compared to untreated cells cultured in phenol‐free medium. Finally, to characterize the effect of estrogen on the CRF receptors, semi‐quantitative PCR and western blot analysis using antibodies specific to CRFR1 and CRFR2 were carried out. The expression of CRFR2 was significantly reduced at both the mRNA and protein level in response to activation of either estrogen receptor (Fig. 3D and E). Expression of CRFR1 appears to be significantly decreased in response to activation of ER*A* and ER*B* at the protein level; however, semi‐quantitative PCR suggests there is only a small decrease at the transcriptional level.

### UCN1 modulates the expression of the estrogen receptors

**Figure 4:**
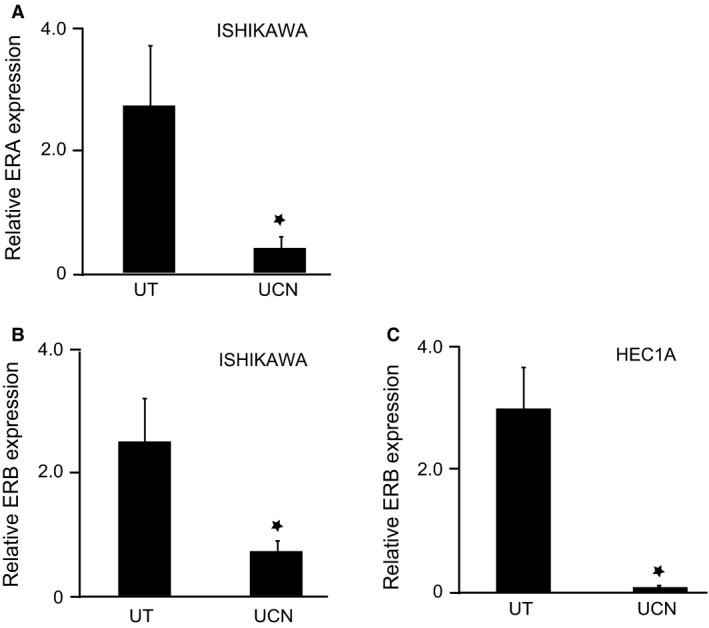
UCN1 down‐regulates ER*A* and ER*B* mRNA expression. (A) QPCR analysis of ER*A* expression in untreated and UCN1‐treated Ishikawa cells. (B) and (C) QPCR analysis of ER*B* expression in untreated cells and UCN1‐treated cells. Each bar represents the mean ± SEM of triplicate samples, and each experiment was repeated three times (*P < 0.05, calculated by Student's t‐test. UT = untreated, UCN = urocortin 1).

Ishikawa and HEC1A cells cultured in normal medium were treated with UCN1. After 24 h incubation, cells were harvested and RNA was isolated as previously described. Quantitative real‐time PCR was performed to assess the expression of ER*A* and ER*B* mRNA in Ishikawa and HEC1A cells. Again, the optimum conditions and primer sequences are described in Table 1. Expression of ER*A* and ER*B* mRNA was determined in untreated cells and UCN1 treated cells. Figure 4 indicates that ER*A* and ER*B* mRNA expression is down regulated in response to UCN1 (*P* < 0.05).

## Discussion

In this study, we have shown that UCN1 significantly suppresses migration of endometrial cancer cells in vitro. Florio et al. 6. previously demonstrated that UCN1 expression is significantly reduced in endometrial cancer compared with healthy endometrium, thus we can infer from this, that loss of UCN1 may promote cancer invasion and metastasis. To the best of our knowledge, only one other study has previously investigated the role of UCN1 in tumor migration. Zhu et al. 11. found that UCN1 promoted cell migration via CRFR1 but conversely suppressed migration via CRFR2 in human hepatocellular carcinoma cell lines. Accordingly, this observation led us to utilize selective antagonists to CRFR1 (CP154526) and CRFR2 (Astressin‐2B), to determine whether the observed effect on cell migration was receptor specific. We subsequently demonstrated that UCN1 reduced endometrial cancer migration via CRFR2, as suppression of migration was completely eliminated by the addition of the CRFR2 antagonist whereas the addition of the CRFR1 antagonist did not reverse UCN1‐induced suppression of migration.

An important question, which arises from the results of these experiments, is: what is the mechanism by which UCN1 reduces endometrial cancer cell migration? Several studies have investigated the role of cytosolic (cPLA_2_) and calcium‐independent (iPLA_2_) phospholipases in cell motility in response to UCN1/CRF 11, 12. Zhu et al. 11. demonstrated that UCN both promoted and inhibited cell migration via differential regulation of cPLA_2_ and iPLA_2._ Specifically, down‐regulation of iPLA_2_ via CRFR2 led to reduced cell motility and up‐regulation of cPLA_2_ via CRFR1 led to increased motility. More recently, Zhu et al. 12. confirmed the pro‐migratory role of CRFR1‐cPLA_2_ signaling pathway in vascular smooth muscle cells. Taken together, these studies suggest that UCN‐mediated suppression of migration could be, at least in part, a result of down‐regulation of iPLA_2_.

Endometrial cancer is a hormone‐driven cancer, with more than 80% of endometrial cancers arising from exposure to estrogen. The “unopposed estrogen hypothesis” proposes that endometrial cancer risk is increased among women who have high circulating levels of bioavailable estrogen and/or low levels of progesterone, and consequently the proliferative effects of estrogen are insufficiently counterbalanced by progesterone 13, 14. This theory is supported by epidemiological data from the Million Women Study, which showed that the use of estrogen‐only hormone replacement therapy (HRT) greatly increased the risk of endometrial cancer, while the use of combined progestagen‐estrogen HRT had a protective effect 15.

The incidence of endometrial cancer is rising in accordance with the obesity epidemic. Obesity is thought to increase endometrial cancer risk by increasing the amount of bioavailable estrogen in the circulation. Following the menopause, when ovarian steroidogenesis ceases, the primary source of estrogen is via aromatization of adrenal androgens in peripheral adipose tissue 16. Additionally, obesity‐related hyperinsulinaemia inhibits the synthesis of sex hormone binding globulin (SHBG), a protein with a high affinity for estradiol and testosterone 17. Collectively, both of these mechanisms increase the amount of bioavailable estrogen than can target the endometrium.

Here, we have demonstrated that UCN1 expression is differentially regulated by estrogen receptor activation. Our results demonstrate that ER*A* activation inhibits UCN1 expression in endometrial cancer, but ER*B* activation has no significant effect on UCN1 transcript expression. Our results are consistent with the findings of several earlier studies, which suggested that estrogen might exert transcriptional regulation of the CRF and UCN1 genes. Previously, Makrigiannakis et al. 18. showed that estradiol inhibited the activity of the CRF promoter in human endometrial adenocarcinoma cell lines. Similar inhibitory effects of estrogen on CRF gene expression have been reported centrally in the paraventricular nucleus of the hypothalamus in ovariectomized rats 19. More recently, Watanabe et al.^20^ demonstrated that subcutaneous injection of estradiol benzoate or an ER*A* agonist significantly suppressed UCN2 mRNA expression levels in the uterus of ovariectomized rats, but administration of an ER*B* agonist had no effect. This suggests that UCN2 is potentially negatively regulated by estrogen through ER*A*.

We have also shown that UCN1 significantly reduced the expression of the estrogen receptors, ER*A* and ER*B*. This firstly suggests that UCN1 may regulate estrogen activity in endometrial cancer cells in an autocrine and paracrine fashion. Secondly, this observation also suggests that loss of UCN1‐mediated inhibition of ER*A* and ER*B*, may lead to increased estrogen activity in endometrial cancer, thus promoting estrogen‐induced proliferation and migration. Previously, Hou et al. 21 demonstrated that overexpression of ER*A* and ER*B* both significantly increased the proliferation, migration, and invasion capacity of endometrial cancer cells in vitro. As such, this raises the question as to whether UCN1′s suppression of migration is actually secondary to UCN1 inhibiting ER*A* and ER*B*.

This study has demonstrated that UCN1 inhibits endometrial cancer cell migration. This suggests that loss of UCN1 in endometrial cancer may promote cancer cell invasion and metastasis. Furthermore, estrogen appears to exert direct transcriptional regulation of UCN1 via ER*A*. Further work is required to understand the mechanisms through which UCN1 suppresses endometrial cancer cell migration, and to characterize the role of UCN2, UCN3, and the CRF‐BP in endometrial cancer.

## Conflict of Interest

PAT is a co‐founder of Karus Therapeutics
